# A latent allocation model for the analysis of microbial composition and disease

**DOI:** 10.1186/s12859-018-2530-6

**Published:** 2018-12-31

**Authors:** Ko Abe, Masaaki Hirayama, Kinji Ohno, Teppei Shimamura

**Affiliations:** 1 Division of Systems Biology, Nagoya University Graduate School of Medicine, 65 Tsurumai-cho, Showa-ku, Nagoya, 4668550 Japan; 2 School of Health Sciences, Nagoya University Graduate School of Medicine, 1-1-20 Daiko-Minami, Higashi-ku, Nagoya, 61-8873 Japan; 3 Division of Neurogenetics, Center for Neurological Diseases and Cancer, Nagoya University Graduate School of Medicine, 65 Tsurumai-cho, Showa-ku, Nagoya, 4668550 Japan; 4 Division of Systems Biology, Nagoya university Graduate School of Medicine, 65 Tsurumai-cho, Showa-ku, Nagoya, 4668550 Japan

**Keywords:** Latent allocation model, Mixture distribution, Metagenomics

## Abstract

**Background:**

Establishing the relationship between microbiota and specific diseases is important but requires appropriate statistical methodology. A specialized feature of microbiome count data is the presence of a large number of zeros, which makes it difficult to analyze in case-control studies. Most existing approaches either add a small number called a pseudo-count or use probability models such as the multinomial and Dirichlet-multinomial distributions to explain the excess zero counts, which may produce unnecessary biases and impose a correlation structure taht is unsuitable for microbiome data.

**Results:**

The purpose of this article is to develop a new probabilistic model, called BERnoulli and MUltinomial Distribution-based latent Allocation (BERMUDA), to address these problems. BERMUDA enables us to describe the differences in bacteria composition and a certain disease among samples. We also provide a simple and efficient learning procedure for the proposed model using an annealing EM algorithm.

**Conclusion:**

We illustrate the performance of the proposed method both through both the simulation and real data analysis. BERMUDA is implemented with R and is available from GitHub (https://github.com/abikoushi/Bermuda).

## Background

Low-cost metagenomic and amplicon-based sequencing has provided a snapshots of microbial communities and their surrounding environments. One of the goals for case-control studies using microbiome data is to investigate whether cases differ from controls in term of the microbiome composition of a particular body ecosystems (e.g., the gut) and which taxa are responsible for any differences observed [1]. (Here, we use the generic term “taxa” to denote a particular phylogenetic classification.) These studies present microbiome data are represented as count data using operational taxonomic units (OTUs). The number of occurrences of each OTU is measured for each sample drawn from an ecosystem, and the resulting OTU counts are summarized for any level of the bacterial phylogeny, e.g., species, genes, family, order, etc. An important feature of these microbiome count data is that it is highly sparse—i.e., a very high proportion of the data entries are zero—which makes analyzing these data difficult.

A common strategy to handle these excessive zeros is to add a small number called a pseudo-count. For example, Xia et al. (2013) [2] applied a logistic normal model to their data, adjusted by a pseudo-count. Although adding a pseudo-count is a simple and widely used strategy, it can add an unnecessary bias to the data. Further, Weiss et al. (2017) [3] noted that there is no clear consensus on how to choose that value. Another common strategy to mitigate the effects of these excessive zeros is to use non-parametric statistical tests. Wagner et al. (2011) [4] described a test statistic that combines the proportion of zeros in the data with the statistics on values other than 0. However, this statistical test can only be used for comparing two taxa. In addition, the test cannot evaluate the co-occurrence relationships between many taxa, or the effect of combination of taxa. Other strategies include modeling excess zeros using probability models [5, 6]. Such an approach is called “zero-inflated" modeling, and directly models the probability of producing excessive zeros. However, zero-inflated models make an implicit assumption that microbial composition is identical among individuals. Thus, such models cannot capture the effects of individual differences in microbial composition.

**Contributions** This article proposes a new probabilistic model, called BERnoulli and MUltinomial Distribution-based latent Allocation (BERMUDA), to address these problems. The contributions of our work are summarized below:

1. BERMUDA is a generative statistical model that allows a set of taxa to be explained by unobserved groups and can be used to find the inherent relationship between taxa and a specific disease and to generate microbiome count data through the model.
2. In BERMUDA, the abundance of each taxon can be viewed as a mixture of various groups, which enables us to describe the differences in bacteria composition between samples.
3. We provide a simple and efficient learning procedure for the proposed model using an annealing EM algorithm that reduces the local maxima problem inherent to the traditional EM algorithm. The software package that implements the proposed method in the R environment is available from GitHub (https://github.com/abikoushi/Bermuda).

We describe our proposed model and algorithm in the “Methods” section. We also provide the efficiency of BERMUDA using synthetic and real data in “A simulation study” and “Results” sections, respectively.

## Methods

### Proposed model

Suppose that we observe a microbial count dataset with disease labels, {(*w*_*nk*_,*y*_*n*_);*n*=1,…,*N,k*=1,…,*K*)}, where *w*_*nk*_ is the abundance of the *k*-th taxon and *y*_*n*_ is a binary outcome such that *y*_*n*_=1 if the *n*-th sample has a certain disease and *y*_*n*_=0 otherwise. Let *w*_*n*_ be the *k*-th row of matrix *W*=(*w*_*nk*_) and $M_{n}=\sum _{k=1}^{K} w_{nk}$ be the total reads count of the *n*-th sample.

**Fig. 1:**
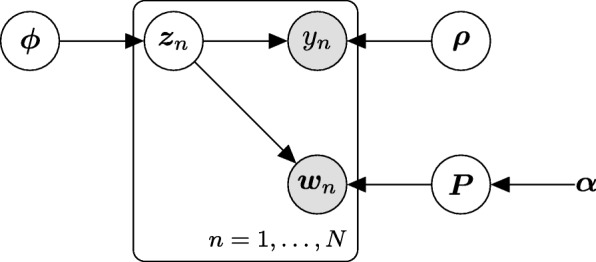
The plate notation for the proposed model

We extract the associations between microbial composition and a specific disease by also supposing that there exist *L* latent clusters that vary with microbial composition and the disease risk. Let *z*_*n*_=(*z*_*n*1_,…,*z*_*nL*_)^*T*^ be an indicator vector such that *z*_*nl*_=1 if the *n*-th sample is in the *l*-th class and *z*_*nl*_=0 otherwise. We then consider the following generative model:

1 $$ \left\{\begin{array}{ll} y_{n}|\boldsymbol{z}_{n},\boldsymbol{\rho} & \sim \text{Bernoulli}\left(\rho^{z_{n1}}_{1} \cdots \rho_{L}^{z_{nL}}\right), \\ \boldsymbol{w}_{n}|M_{n},\boldsymbol{z}_{n}, \boldsymbol{P} & \sim \text{Multinomial}\left(M_{n}, \boldsymbol{z}_{n}^{T} \boldsymbol{P}\right), \\ \boldsymbol{z}_{n}|\boldsymbol{\phi} & \sim \text{Multinomial}(1,\boldsymbol{\phi}),\\ \boldsymbol{p}_{l}|\boldsymbol{\alpha} & \sim \text{Dirichlet}(\boldsymbol{\alpha}), \end{array}\right.   $$

where *ρ*=(*ρ*_1_,…,*ρ*_*L*_)^*T*^ is the probability of developing a certain disease, *P*=(*p*_*lk*_) (*l*=1,…*L*) is an *L*×*K* matrix of the appearance probability of taxa, *p*_*l*_ is the *l*-th row vector of matrix *P*, *ϕ*=(*ϕ*_1_…,*ϕ*_*L*_)^*T*^ is a vector of each component’s mixing ratios, and *α*=(*α*_1_,…,*α*_*K*_)^*T*^ is a vector of the hyperparameters of the Dirichlet prior distribution. Figure 1 displays the plate notation for the proposed model. The gray node represents an observed variable and the white node represents an unobserved variable; the latent variable *z*_*n*_ affects both *y*_*n*_ and *w*_*n*_.

If the latent variable *z*_*n*_ is given, the complete likelihood of this model is represented by the following formula:

2 $$ {\begin{aligned} \prod\limits_{n=1}^{N}f&\left(y_{n},\boldsymbol{w}_{n},\boldsymbol{z}_{n}|\boldsymbol{P},\boldsymbol{\rho},\boldsymbol{\phi}\right) \\ &= \prod\limits_{n=1}^{N} \prod\limits_{l=1}^{L} \phi_{l}^{z_{nl}} \left\{\rho_{l}^{y_{n}} \left(1-\rho_{l}\right)^{1-y_{n}}\right\}^{z_{nl}} \left(\frac{\left({\sum\nolimits}_{k=1}^{K} w_{nk}\right)!}{w_{n1}! \cdots w_{nK}!} \prod\limits_{k=1}^{K}\left(p_{lk}^{w_{nk}}\right)^{z_{nl}} \right).  \end{aligned}}  $$

The posterior distribution is then proportional to:

3 $$\begin{array}{*{20}l} \exp\left(\sum\limits_{n=1}^{N} \log f\!\left(y_{n},\boldsymbol{w}_{n},\boldsymbol{z}_{n}|\boldsymbol{P},\boldsymbol{\rho},\boldsymbol{\phi}\right) + \!\sum\limits_{l=1}^{L} \sum\limits_{k=1}^{K}\! \left(\alpha_{k} - 1\right)\log p_{lk}\right).  \end{array} $$

### Parameter estimation

We find the maximum a posteriori probability (MAP) estimators, using an annealing EM (AEM) algorithm [7]. One advantage of using an AEM algorithm is that it reduces the local maxima problem from which the traditional EM algorithm suffers.

In the E-step, using the inverse temperature 0<*β*≤1, we calculate

4 $$\begin{array}{*{20}l} z_{nl}^{(i+1)} = \frac{ f\left(y_{n},\boldsymbol{w}_{n},z_{nl} |\boldsymbol{P}^{(i)}, \boldsymbol{\rho}^{(i)}, \boldsymbol{\phi}^{(i)}\right)^{\beta} }{{\sum\nolimits}_{z_{nl}} f\left(y_{n}, \boldsymbol{w}_{n},z_{nl} |\boldsymbol{P}^{(i)},\boldsymbol{\rho}^{(i)},\boldsymbol{\phi}^{(i)}\right)^{\beta}}.  \end{array} $$

To simplify the explanation, we set *γ*=*α*_*k*_−1. From the logarithm of (3), in the M-step, we update the parameters using:

5 $$\begin{array}{*{20}l} \phi^{(i+1)}_{l} &= \frac{1}{N}\sum\limits_{n=1}^{N} z_{nl}^{(i+1)},  \end{array} $$

6 $$\begin{array}{*{20}l} \rho^{(i+1)}_{l} &= \frac{{\sum\nolimits}_{n=1}^{N}z_{nl}^{(i+1)} y_{n}}{{\sum\nolimits}_{n=1}^{N} z_{nl}^{(i+1)}}, \end{array} $$

7 $$\begin{array}{*{20}l} p^{(i+1)}_{lk} &= \frac{{\sum\nolimits}_{n=1}^{N}z_{nl}^{(i+1)} w_{nk} + \gamma}{{\sum\nolimits}_{n=1}^{N} z_{nl}^{(i+1)} M_{n} + K\gamma}. \end{array} $$

If *γ*=0, MAP estimators are equivalent to maximum likelihood estimatos (MLEs).

The procedure of BERMUDA is then summarized as follows:

1. Set *β*.
2. Arbitrarily choose an initial estimate *P*^(0)^, *ϕ*^(0)^ and *ρ*^(0)^. Set *i*←0.
3. Iterate the following two steps until convergence:
  - E-step: Compute $z^{(i+1)}_{nl}$ from (4).
  - M-step: Compute *P*^(*i*+1)^, *ϕ*^(*i*+1)^ and *ρ*^(*i*+1)^ from (5), (6) and (7). Set *i*←*i*=*i*+1.
4. Increase *β*.
5. If *β*<1, repeat from step 3; otherwise stop.

Let $\hat{\boldsymbol{\phi}}$, $\hat{\boldsymbol{\rho}}$ and $\hat{\boldsymbol{P}}$ be MAP estimators of *ϕ*, *ρ* and *P*. If given *w*_*n*_ and the estimators, we can evaluate the probability that the *n*-th sample has the target disease. The conditional probability is given by

8 $$\begin{array}{*{20}l} \tilde \rho_{n} &= \Pr\left(y_{n}=1|\boldsymbol{w}_{n}, \hat{\boldsymbol{P}}, \hat{\boldsymbol{\rho}}, \hat{\boldsymbol{\phi}}\right)  \\ &= \frac{\Pr\left(y_{n}=1, \boldsymbol{w}_{n} |\hat{\boldsymbol{P}}, \hat{\boldsymbol{\rho}}, \hat{\boldsymbol{\phi}}\right)}{\Pr\left(\boldsymbol{w}_{n} |\hat{\boldsymbol{P}}, \hat{\boldsymbol{\rho}}, \hat{\boldsymbol{\phi}}\right)} \\ &= \frac{ {\sum\nolimits}_{z_{nl}} f\left(y_{n}=1,\boldsymbol{w}_{n},z_{nl} |\hat{\boldsymbol{P}}, \hat{\boldsymbol{\rho}}, \hat{\boldsymbol{\phi}}\right)}{{\sum\nolimits}_{z_{nl}} {\sum\nolimits}_{y_{n}} f\left(y_{n},\boldsymbol{w}_{n},z_{nl} |\hat{\boldsymbol{P}}, \hat{\boldsymbol{\rho}}, \hat{\boldsymbol{\phi}}\right)}.  \end{array} $$

The advantage of using the Dirichlet prior distribution is that we can evaluate the abundance of the taxa whose abundance is exactly zero.

The *n*-th sample is then classified into the *l*-th class that maximizizes the conditional probability given by

9 $$\begin{array}{*{20}l} \hat{z}_{nl} = \frac{ f\left(y_{n},\boldsymbol{w}_{n},z_{nl} |\boldsymbol{P}^{(i)}, \boldsymbol{\rho}^{(i)}, \boldsymbol{\phi}^{(i)}\right) }{{\sum\nolimits}_{z_{nl}} f\left(y_{n}, \boldsymbol{w}_{n},z_{nl} |\boldsymbol{P}^{(i)},\boldsymbol{\rho}^{(i)},\boldsymbol{\phi}^{(i)}\right)}.  \end{array} $$

In fitting the model, it is important to choose an appropriate number for *L*. In this article, we use cross-validation to choose *L*. From (8), we can evaluate the probability that the *n*-th sample has the target disease. We can then evaluate the log-loss function represented by:

10 $$\begin{array}{*{20}l} LL=-\sum\limits_{j=1}^{J}{\left(y_{j}\log\left(\tilde \rho_{j}\right) + \left(1 - y_{j}\right)\log\left(1 - \tilde \rho_{j}\right)\right)},  \end{array} $$

where *J* is an arbitrarily chosen subsample size for the validation data. We then select an *L* which minimizes (10) in this analysis.

## A simulation study

In this section, we generated synthetic data and evaluated the performance of our method in order to gain insights into the accuracy of the parameters estimated using the proposed model. A simulation study was conducted as follows. An i.i.d. sample is generated by (1) where we set *N*=700, *M*_*n*_=10000, *L*=7, *γ*=10^−9^, *ϕ*=(1/7,…,1/7)^*T*^, and *ρ*=(0,3,0.4,…,0.9)^*T*^. *P* is chosen by a standard Dirichlet random number. We estimated the parameters from 10,000 replicates of the experiment.

**Fig. 2:**
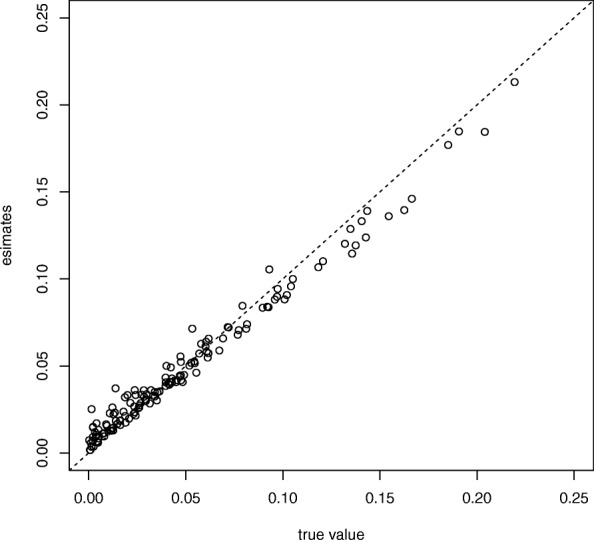
The comparison of true *P* and mean of $\hat{\boldsymbol{P}}$

Table 1 shows the mean and standard error (se) of the estimates for *ρ* and *ϕ* using the proposed method. It can be observed that the estimates are unbiased to the order of 1/100. Figure 2 shows the relationship between estimates and true *P* in this simulation. In this figure, the points are arranged diagonally, implies that the estimator is unbiased. The overall accuracy of classification by $\hat {z}_{nl}$ (9) is 0.87.

**Table 1:** The mean and se of $\hat{\boldsymbol{\rho}}$ and $\hat{\boldsymbol{\phi }}$

Cluster	1	2	3	4	5	6	7
*ρ*	0.30	0.40	0.50	0.60	0.70	0.80	0.90
Mean	0.30	0.40	0.50	0.60	0.70	0.80	0.90
se	0.05	0.05	0.05	0.05	0.05	0.04	0.03
*ϕ*	0.14	0.14	0.14	0.14	0.14	0.14	0.14
Mean	0.14	0.14	0.14	0.14	0.14	0.14	0.14
se	0.01	0.01	0.01	0.01	0.01	0.01	0.01

## Results

### Parkinson’s disease data

We first seek to identify the gut dysbiosis in relation to the development of Parkinson’s disease (PD), which is thought to be associated with intestinal microbiota. We analyzed intestinal microbial data for PD cases and controls in three different countries. Scheperjans et al. (2015) [8], Hill-Burns et al. (2017), [9], Hopfner et al. (2017) [10] and Heintz et al. (2018) [11] conducted case-control studies by sequencing the bacterial 16S ribosomal RNA gene in Finland, the USA, and Germany, respectively.

The OTUs are then mapped to the SILVA taxonomic reference, version 132 (https://www.arb-silva.de/) and the abundances of genus-level taxa are calculated. We focused on the top 20 genera in terms of sample mean of normalized abundance *w*_*nk*_/*M*_*n*_ for 336 PD cases and 277 controls.

**Fig. 3:**
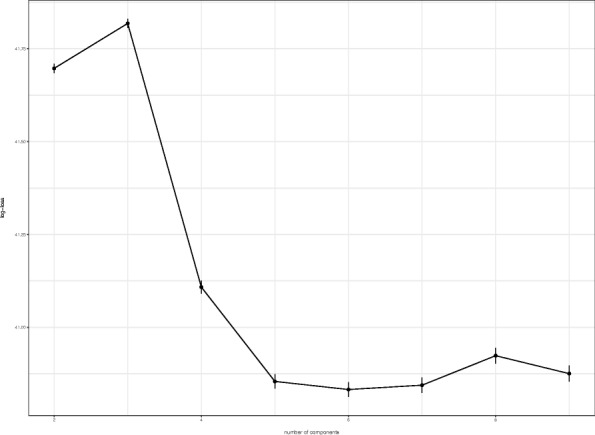
The behavior of the log-loss functions given by different numbers of components *L*. The error-bars indicate standard error

We set *γ*=10^−9^, which is equivalent to giving a weakly informative prior. The number of components *L*=6 is selected using 10-fold cross-validation (Fig. 3). To ensure the stability, we iterated the cross validation 10,000 times and used the mean of log-loss functions. Figure 3 shows the log-loss functions for different numbers of the components *L*.

**Fig. 4:**
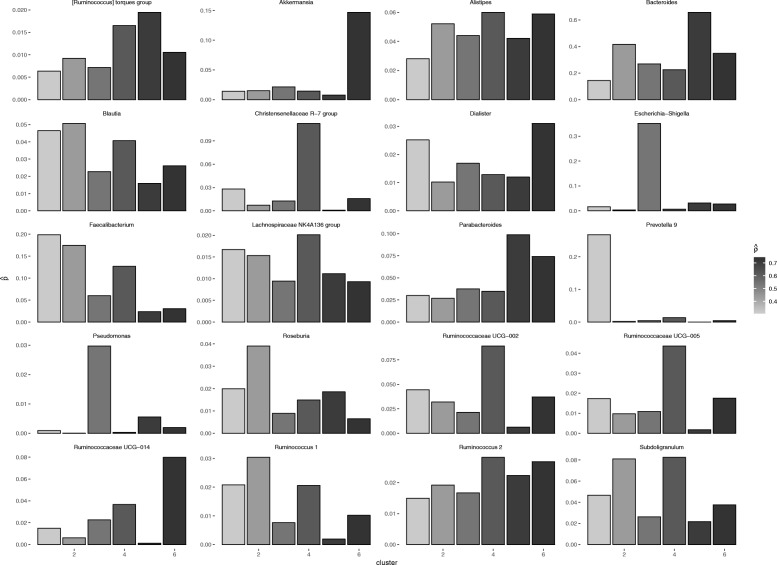
The appearance probability of the 20 genera ($\hat{\boldsymbol{P}}$)

Figure 4 presents the estimated appearance probabilities of the 20 genera. The clusters are sorted by estimated PD risk $\hat{\boldsymbol{\rho}}$ (Table 2). As displayed Fig. 4, the distribution of *Prevotella* is quite distinctive, being concentrated in the low-risk cluster of PD. *Faecalibacterium* also tends to be higher in the low-risk cluster. In contrast, *Akkermansia* is concentrated in the high-risk cluster.

**Table 2:** The estimated disease risk ($\hat \rho _{l}$) within each cluster

	1	2	3	4	5	6
1	0.31	0.43	0.54	0.62	0.71	0.73

### Zeller’s colorectal carcinoma data

Next, we investigate the identification of gut dysbiosis associated with the development of colorectal cancer (CRC). Zeller et al. (2014) [12] studied gut metagenomes extracted from 157 persons, 91 of whom are CRC patients and 66 are controls. The data are available as an R package “curatedMetagenomicData” (https://github.com/waldronlab/curatedMetagenomicData). In the analysis, we used the abundance of order-level taxa.

**Fig. 5:**
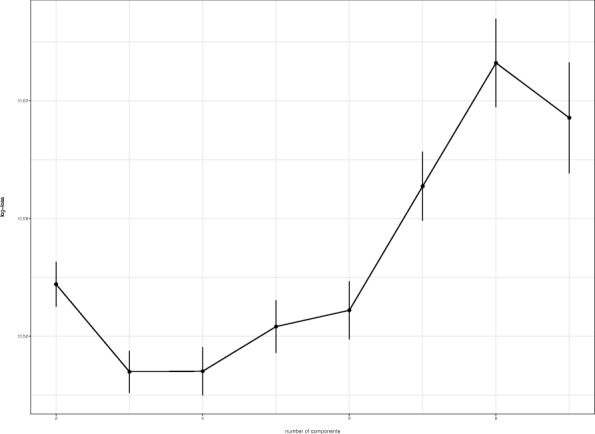
The behavior of the log-loss functions given by different numbers of components *L*. The error-bars indicate standard error

While training the model, we set *γ*=10^−9^. The number of components *L*=3 was selected using 10-fold cross-validation. To ensure the stability, we iterated the cross validation 10,000 times and used the mean of log-loss functions. Figure 5 shows that log-loss functions for different numbers of the components, *L*. The clusters are sorted by the estimated CRC risk $\hat{\boldsymbol{\rho }}$.

**Fig. 6:**
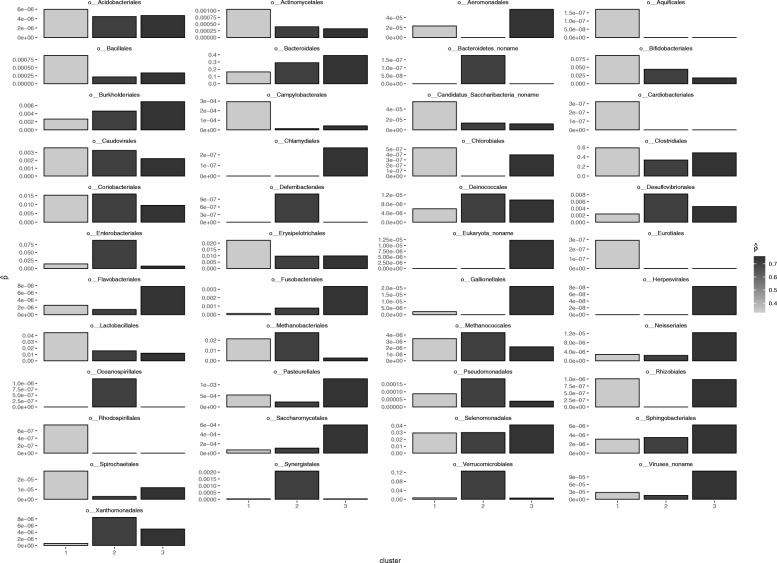
The appearance probability of the 45 orders ($\hat{\boldsymbol{P}}$)

Figure 6 presents the estimated appearance probabilities for each cluster. Previous studies showed that *Fusobacterium* flourishes in colorectal cancer cells [13]. Figure 6 shows that the abundance of *Fusobacteriales* is positively correlated $\hat{\boldsymbol{\rho }}$. We also observe bacteria, such as *Bacteroides* and *Chlamydiales* with monotonically increasing abundance. This result indicates that BERMUDA can be a valuable tool for discovering new disease-related bacteria.

## Discussion

We evaluated the accuracy of parameter estimation using the simulated data. Table 1 and Fig. 2 shows that the proposed method can produce reasonable estimates and classify samples into true groups.

We also applied BERMUDA to the real metagenomic sequencing data in order to identify the associations between the gut microbiota and PD. We compared the results of BERMUDA with those of previous studies. Petrov et al. (2016) [14] reported that the gut microbiota of PD patients contained high levels of *Christensenella*, *Catabacter*, *Lactobacillus*, *Oscillospira*, and *Bifidobacteriumm*, and the control cluster was characterized by increased content of *Dorea*, *Bacteroides*, *Prevotella*, and *Faecalibacterium*. In family level analysis, Hill-Burns et al. (2017) [9] reported PD patients contained high levels of *Bifidobacteriaceae*, *Lactobacillaceae*, *Tissierellaceae*, *Christensenellaceae* and *Verrucomicrobiaceae* and low levels of *Lachnospiraceae*, *Pasteurellaceae*. *Scheperjans* et al.(2015) [8] reported PD patients contained high levels of *Lactobacillaceae*, *Verrucomicrobiaceae*, *Bradyrhizobiaceae* and *Ruminococcaceae* and low levels of *Prevotellaceae* and *Clostridiales Incertae Sedis IV*. *Akkermansia* belongs in *Verrucomicrobiaceae*. Of the *Verrucomicrobiaceae*, it has been suggested that *Akkarmansia* may be related to PD. BERMUDA revealed *Prevotella*, *Faecalibacterium*, and *Akkermansia* associated with PD, which were commonly found in several studies. Thus, the analysis with real data demonstrates that the proposed method can identify the connection between the gut microbiota and PD, with the results are strongly supported by the previous PD research.

## Conclusion

We proposed the new probabilistic model BERMUDA to analyze the relationship between microbiota and a specific diseases. Although the existing approaches tend to underestimate individual differences in microbial composition, BERMUDA can take into account these differences and identify combinations of taxa rather than single taxa in the analysis of association with a specific disease risk. We demonstrated the applicability of BERMUDA to microbial analyses with simulation and real data. We expect that BERMUDA can be efficiently applied to studies that seek for an association between gut dysbiosis and a specific disease.
